# Influence and Relationship of Pain on Lumbar Biomechanics in a Young Adult Population with Non-Specific Low Back Pain

**DOI:** 10.3390/sports12070190

**Published:** 2024-07-11

**Authors:** Sagrario Pérez-de la Cruz

**Affiliations:** Department of Nursing, Physiotherapy and Medicine, University of Almería, 04120 Almería, Spain; spd205@ual.es; Tel.: +34-90214574

**Keywords:** assess, low back pain, mobility, quality of life, sport

## Abstract

The therapeutic actions indicated for low back pain, in addition to physiotherapy, include mobilization of the affected segment, as it is assumed that a loss of mobility may contribute to a patient’s pain. The aim of this study was to investigate the influence of back pain on the degrees of spinal mobility in young adults. Eighty-six volunteers participated in the study. Fingertip-to-floor distance, Schöber’s test, the fingertip-to-floor lateral flexion test, GHQ-12, the Fear-Avoidance Beliefs Questionnaire and the STarT Back Screening Tool were used. There were statistically significant differences between the two groups (pain and no pain) in degrees of spinal flexion (Schöber’s test and side flexion) showing greater mobility in the group with pain. However, the group with low back pain showed less rotational mobility. The presence or absence of back pain had an impact on the individual’s sporting practice and perception of pain, and they were able to carry out their sporting activities normally. Young adults with idiopathic low back pain showed some statistically significant differences in relation to the mobility of the spine in the different planes of movement (flexion and side flexion), conditioning their quality of life and sports practice.

## 1. Introduction

Low back pain (LBP) is a highly prevalent and disabling musculoskeletal condition [1]. For many people, LBP becomes a chronic condition characterized by persistent pain and limitations in functional activities [2,3], reducing their quality of life and ability to practice sports. The concept of non-specific LBP was introduced to designate LBP for which no identifiable cause could be found on examination or imaging studies [1]. Around 20% of people who have suffered from acute LBP develop chronic LBP when symptoms persist after three months [4].

Complaints of LBP are one of the most frequent reasons for medical consultation, representing a major economic burden for the Spanish National Health system every year, with considerable personal and occupational costs for the patient [5,6].

The treatment of LBP involves a variety of intervention strategies, including surgery, pharmacological treatment and non-surgical interventions [7]. In recent years, numerous randomized controlled trials (RCTs) have been published and summarized in systematic reviews. Most of these systematic reviews focus on the efficacy of a single intervention and describe efficacy in different types of LBP. Physical medicine and rehabilitation interventions include exercise therapy [8], behavior-based school programs, transcutaneous electrical nerve stimulation (TENS), superficial heat or cold, low level laser therapy (LLLT), massage, behavioral therapy, lumbar supports, traction and multidisciplinary rehabilitation [9,10]. Another therapeutic approach indicated for the treatment of this pathology is proprioceptive work and postural control of the affected region through therapeutic exercise [11].

Proprioception is a key element of the somatosensory system that facilitates the coordination of sensory information, central analysis and the execution of movements to maintain balance. A systematic review [12] found that proprioception is more impaired in people with chronic low back pain than in healthy people in the control group. Alterations in lumbar spine movement may be caused by proprioceptive deficits, which could be a factor in low back pain [12,13]. Different types of therapeutic exercises are used in health care for people with low back pain, such as proprioceptive therapeutic exercise. Core stabilization exercises, based on motor learning, emphasize the simultaneous activation of the transverse abdominal and multifidus muscles in the lumbar region. Muscle strengthening exercises are commonly used to treat patients with low back pain because these deep stabilizing muscles connect to the thoracolumbar fascia, creating rigidity in the lower back by increasing pressure on the abdomen and providing stability to each segment of the spine, reducing pain and physical disability [14,15]. Strengthening programs can increase gamma motor activity, improve central motor control or generate a mixture of central and spindle mechanisms [16].

For the treatment of LBP, mobility exercises are believed to provide a greater range of joint amplitude, which could be one of the indications for treating LBP, considering that lack of mobility may be one of the reasons for the appearance of pain. Hence, this study proposes to compare two groups of young adults, one with back pain and the other without, and to compare the range and degree of mobility, in the assumption that pain limits mobility, and, therefore, to prescribe mobilization activities in patients with back pain, due to the loss of range related to their LBP process.

The aim of this study was to investigate the influence of LBP on the degree of mobility of the spine in young adults with and without LBP. The hypothesis is that people with non-specific LBP present mobility restrictions affecting certain ranges of spinal movement, which would support the proposal of physical activity for total recovery from non-specific LBP in adulthood.

## 2. Materials and Methods

This descriptive study was carried out in collaboration between the University of Almeria and the Spanish National Police School.

The sample consisted of 86 participants (46 men and 40 women) who met the following inclusion criteria: legal age (18 years and over), voluntary participation in the study, signing of the informed consent form and practicing a sporting activity in their daily life (not at a competitive or professional level). There was a differentiation between people who had presented an episode of non-specific LBP in the last three months and those who had not (the sample was divided into two distinct groups). The medical service of the participating centers corroborated the diagnosis of chronic non-specific LBP (with no diagnosable underlying pathology) in the final study sample (medical examination and radiological tests). The diagnosis was determined in a previous medical examination, and this service determined whether they could participate, and to which group the subject would belong based on the characteristics shown in the medical examination carried out at the time of admission to the center. However, people with back surgery which entailed a limitation to their daily life were considered as causes for exclusion.

All participants were informed of the purpose of the study, of their autonomy and of the absolute confidentiality of their responses. Their participation was voluntary. Those who agreed to participate after reading the information sheet were asked to sign the informed consent.

A questionnaire was used which included a series of demographic variables, such as sex, age, level of studies and time spent practicing sport. Ethical approval for this study was granted by the Bioethics Committee of the University of Almeria (UALBIO2020/022). This study was conducted between March and September 2023.

Scales:

Fingertip-to-floor distance: To carry out the fingertip-to-floor distance test towards anterior flexion (also known in the literature as the fingertip-to-floor test (FFD) or toe-touch test), the studies by Robinson et al. and Perret et al. [17,18] were used as a reference, which demonstrate its validity and reliability. This test assesses the global mobility of the spine. The initial position of the subject is in a standing position, barefoot, with the upper limbs relaxed alongside the body, the eyes looking straight ahead and the feet hip-width apart. The command for the patient to perform the test is to “bend forward as far as possible, keeping your knees, arms and fingers fully extended and trying to touch the floor with your hands”. The patient is instructed to perform the test twice, once as a test, and the next time the measurement is taken [17]. The physiotherapist measures the distance in centimeters between the ground and the third finger.

Schöber’s test: This test is performed with the patient standing barefoot with feet 30 cm apart. A point is then marked 5 cm below the spinous process S2 and a point 10 cm above it. Measure the distance between the two points, which should be 15 cm. The patient then flexes as far as possible, keeping the legs fully extended, and the distance is measured again. The difference between the two measurements indicates the amount of flexion [17,19].

The fingertip-to-floor lateral flexion test (also known in the literature as the fingertip-to-floor lateral flexion test (FFD-L) or lateral bending) was based on the descriptions by Norkin and White (2006) [20] and Quack et al. (2007) [21]. The test is used to assess the global mobility of the spine for side flexion. The starting position for the subject is standing barefoot, with the upper limbs relaxed alongside the body, the eyes facing forward and the feet hip-width apart. The physiotherapist measures the vertical distance between the tip of the third finger and the ground in centimeters. The test is performed bilaterally. 

The range of axial rotational movement (to the right and left) is also assessed quantitatively. To estimate the degree of spinal rotation, the pelvis should be held in a fixed position by the examiner’s hands. The patient is instructed to rotate to the right and then to the left, while holding the scapula in a fixed position. 

GHQ-12: The 12-item General Health Questionnaire [22] is used to measure mental health. This scale consists of 12 propositions that must be answered by choosing one of the possible answers given to the participant on a Likert-type scale. This test is used as a screening tool for detecting recent mental disorders, as the examiner must ask about the existence of any discomfort or disorder and the participant’s health over the last two weeks. The total score is calculated by adding the scores obtained in all the statements of the scale (the higher the score indicates a lower level of mental health).

The Fear-Avoidance Beliefs Questionnaire (FABQ) is a widely used questionnaire focusing on pain-related anxiety in patients with LBP. The FABQ can be used to determine the extent to which chronic LBP is affected by physical activity and work components. This version consists of 16 questions divided into two subscales in which reference is made to how physical activity (first 5 questions) and work activity (last 11 questions) affect LBP. The subject must provide a score ranging from 0 (totally disagree) to 6 (completely agree) for each of the questions according to how the person perceives their situation in relation to the work and physical activity aspect. The higher the score, the higher the level of fear-avoidance. High scores indicate a high level of fear beliefs that lead to the avoidance of physical activities [23].

The STarT Back Screening Tool (SBST) is a simple prognostic questionnaire that helps the health professional to identify modifiable risk factors (biomedical, psychological and social) for disability caused by back pain. Moreover, it is a validated scale that has been shown to be comprehensible and adapted to the Spanish population [24]. It contains 9 items that evaluate the physical factors (pain in the legs and other locations) and psychosocial factors (discomfort, catastrophism, fear, anxiety and depression) that have been identified as indicators of worse prognosis for patients. For the scoring, a dichotomous score (0: no; 1: yes) is made, which is scored from 0 to 9. If the score is 3 or less, the person is considered low risk. If the score is less than or equal to 3, the patient is considered medium risk, whereas a score greater than or equal to 4 means that the patient is considered high risk.

Statistical analysis: A descriptive statistical analysis of the variables included in the study was performed. Quantitative variables were expressed as the mean ± standard deviation (SD). The normality of the data was tested using the Kolmogorov–Smirnov test. Comparison of means was performed by Student’s *t*-test or the Mann–Whitney test for independent samples. The correlation of quantitative variables was carried out by means of multiple linear regressions. For the study of reliability associated with continuous variables (spine movements), the intraclass correlation coefficient (ICC) was used. In each of these analyses, the *p*-value was observed to determine whether it was possible to reject the null hypothesis (*p*-value < 0.05) or whether it was not possible to reject this hypothesis due to a lack of significance.

For the statistical analysis of the data, the statistical software IBM SPSS version 20 for Windows was used.

## 3. Results

The final study sample consisted of 86 participants, of which 46.5% (n = 40) were women and 53.5% (n = 46) were men, aged between 20 and 41 years with an average age of 28.4 years (SD = 4.2). The totality of the sample had a medium-high level of education and the average time spent playing sports per week was 5.30 h per week (SD = 2.7). According to the participation group, 36% (n = 31) had no pain whereas 64% had pain (n = 55).

For both groups, axial mobility measurements were taken in the different planes of space. Table 1 shows the values obtained, both in the sample with and without non-specific LBP.

Table 2 shows the values obtained in the tests concerning quality of life, pain perception and perceived level of physical activity in the participants of both groups.

To determine the possible effect of the pain variable and the results of the General Health Questionnaire (GHQ), the Fear-Avoidance Beliefs Questionnaire (FAB_TOTAL) and the Start-Back total according to the Schöber’s test for flexion and extension, multivariate linear regression models were calculated, and the results are shown below.

In the models for the Schober’s test (Table 3), the model for flexion was statistically significant (*p* = 0.041), with patients with pain showing greater flexion than patients without pain. Also, in the case of the GHQ, high levels of GHQ were associated with low levels of flexion, and in the case of the FAB, high levels of FAB were related to high levels of flexion. In the case of extension, none of the variables showed significant effects.

In the analysis of side flexion (Table 4), both on the right and on the left, the pain variable had a statistically significant effect, with subjects with pain exhibiting decreased side flexion compared to subjects without pain.

In the case of rotation (Table 5), rotation towards the right showed a statistically significant effect in the group with LBP, with subjects with pain showing the greatest rotation compared to subjects without pain.

## 4. Discussion

The aim of this study was to analyze the differences in spinal mobility between adult subjects with non-specific LBP and healthy subjects. The results have shown that the values of the Schober’s test in patients with pain are associated with a greater degree of forward flexion compared to patients without pain. However, no significant effects were found for the variables in relation to spinal extension. The rotation assessment revealed that patients with pain showed less range of motion compared to asymptomatic patients.

Any degenerative process at the intervertebral level has been shown to reduce the range of joint mobility [25,26,27] and there is a limitation of mobility in more mobile regions of the spine (lumbar area), together with an impairment of pelvic rotation around the hip, which may increase the risk of injury to the intervertebral disc and ligamentous complex [28]. These statements were based on the Schober’s test and the fingertip-to-floor test [29]. The modified Schober’s test is used to assess the specific mobility of the lumbar spine during anterior trunk flexion [29], and the fingertip-to-floor test records the range of motion of the lumbar spine in the frontal plane, based on side flexion to both sides (right and left). The results of the studies related to these diagnostic procedures have shown that the values in both tests were lower in the group with LBP (*p* < 0.005) compared to the population of healthy subjects [30,31,32,33,34,35]. Numerous studies have investigated the relationship between joint amplitude and LBP. Authors such as Mellor et al. [36] determined that the range of mobility was lower in subjects with non-specific LBP compared to healthy subjects; however, a significant correlation was observed between the values obtained in the mobility tests and the presence of degenerative signs and alterations at the level of the intervertebral disc at the lumbar level. Only a weak correlation was found between structural changes at the L4–L5 level and side flexion. The author justified these facts by the influence of external factors, such as extensibility and/or the stiffness of the surrounding tissue and functional impairment of other underlying structures during the anterior trunk flexion movement [36]. Another study that reviewed this issue of axial mobility determined how decreased lumbar mobility during the anterior flexion movement was a protective response adopted by subjects with non-specific LBP to pain in the face of movement [37]. The action of the lumbar erector spinae muscle in pain-causing movement was determined, including its activation during pain-free periods, and it was observed that the amplitudes of side flexion were also compromised [38]. In addition, other studies related to axial mobility and pain, such as that carried out by Hirata et al. in 2015 [39], describe the protective strategy used by the human body to limit the lumbar movement generated by the excessive activity of the trunk muscles in the face of pain or to prevent the onset of pain. The spinal erectors, anterior rectus and external oblique were activated, and even the transformation of activation patterns from phasic to tonic was demonstrated, with the aim of limiting the movement of the spinal column. Initially, this results in a decrease in motor activation and a reduction in pain; however, in the medium and long term, it alters the correct functionality of the spine, thus demonstrating the relationship between pain, increased muscle activity and decreased mobility at the lumbar level [39].

Conversely, other authors have described how lumbar and pelvic mobility in subjects with LBP is similar to that found in healthy subjects, suggesting that the reason for this decrease in joint range is due to the shortening or stiffness that these patients present at the level of the hamstring musculature [40]. This hypothesis is not currently accepted by a large part of the scientific community, as other studies argue that the shortening of the hamstring musculature has no direct influence on pelvic mobility [41], observing that there is no direct relationship between pelvic mobility and the lower back. They argue that this is associated with an alteration of the neuromuscular pattern associated in people with LBP [42,43]. In the present study, the results obtained are similar to those reported by Zemková E. et al. [40]. The reason for these results may be due to the type of sample selected. In the aforementioned studies, healthy subjects were analyzed, however, there was no indication regarding what type of physical activity the subjects performed or whether they were sedentary. In our sample, an inclusion criterion was the frequent practice of physical activity, not on a professional basis. This may explain why, despite the presence of non-specific LBP (we cannot affirm that they previously presented intervertebral disc degeneration or physical anomalies), sports practice leads to the maintenance (or even improvement) of joint mobility. Another factor that may influence the divergence of results with other related studies may be the level of pain tolerance of each person. The practice of sport implies that the execution of the motor gesture involves nociceptive perceptions that must be tolerated by the subject, leading to an increase in their pain perception threshold that is different from that of a healthy, non-active subject.

Lack of spinal flexibility can be considered a cause of disability in people with non-specific LBP, which in turn is related to a decrease in their quality of life. This can provoke anxiety and mental health problems in young adults with back pain and has been tested in the study sample. Both groups (pain versus no pain) showed similar results, with no significant differences. The results shown in the GHQ scale (which reflects the state of mental health of an individual in the face of physical problems) obtained results which associate a high level of suffering problems in people who obtained a low range of spinal flexion; however, these differences were not significant compared to the asymptomatic sample. The same interpretation was obtained in the FABQ scale, which focused on anxiety related to pain in patients with LBP, which can be used as a tool to determine the extent to which pain can be affected by physical activity and work. It is worth noting that the young adults in the sample are active people who practice sports on a regular basis.

The results show that the range of spinal flexion influences values related to anxiety, although the findings are not significant. Thus, one may suspect that in young subjects with LBP, regardless of the origin, there is a fear of developing certain movements or sporting gestures that may reproduce the injury mechanism that produces pain, and, consequently, this may facilitate situations of restricted mobility and disability [44]. Different authors have shown that fear of pain and of the recurrence of their pathology has a strong potential at a mental and physical level for modulating muscle activation in the area involved [39,45]. However, this fact was not a determining factor for the sample in this study, and no considerable differences were found between the values shown in the healthy population and those with pathology.

In relation to the practice of sports in this type of population, the results obtained on the IPAC scale and the SBST show that the practice of sport at a non-professional level is not influenced by the perception of pain. Thus, both subjects with and without pain share similar sports practice regimes, without impacting the perception of discomfort or pain during the execution of the sporting gesture. However, it has been shown that practicing sports or maintaining motor activity has a positive influence on one’s health, helping to maintain quality of life [5,8,9].

This study has a number of limitations that should be considered when extrapolating the results to the whole population. Future studies should consider recruiting a more homogeneous sample in relation to the sport or physical activity practiced, as different sporting gestures may vary the degree of spinal flexibility. Also, future studies should test these results on a larger sample in order to corroborate the results obtained in this study with greater accuracy and reliability.

## 5. Conclusions

The findings of this study reveal no significant differences between the spinal mobility ranges of young adults who report LBP and those without pain. There are minimal differences in values such as flexion, side flexion and rotation, however, this does not imply a limitation in their activities of daily living, sports practice or perceived quality of life.

These results may indicate that in the protocol for the recovery of back injuries for this type of population, it is not necessary to propose activities for the recovery of movement at the axial level, since there are no differences compared to healthy individuals.

## Figures and Tables

**Table 1:** Values obtained in the spinal column amplitude tests.

	Pain, Mean (SD)		Average Differences	Student’s *t* Test		*d*
	No	Yes		*t (52)*	*p*-Value	
Toe-ground clearance (cm)	5.39 (8.89)	4.22 (6.22)	1.17	0.71	0.477	0.16
Schöber flexion (cm)	14.84 (0.77)	15.35 (0.93)	−0.51	−2.57	0.012	−0.58
Schöber extension (cm)	7.58 (0.72)	7.72 (0.90)	−0.14	−0.73	0.469	−0.16
Right side flexion (cm)	48.55 (4.89)	46.39 (4.08)	2.16	2.19	0.031	0.49
Left side flexion (cm)	49.44 (4.95)	47.30 (4.20)	2.14	2.12	0.037	0.48
Right rotation (degrees)	44.36 (15.55)	49.85 (14.60)	−5.49	−1.64	0.106	−0.37
Left rotation (degrees)	44.71 (16.56)	51.24 (14.10)	−6.53	−1.93	0.056	−0.43

cm: centimeters.

**Table 2:** Values obtained on the scales.

	Pain, Mean (SD)		Average Differences	Student’s *t* Test		*d*
	No	Yes		*t (52)*	*p*-Value	
FAB_Total	6.23 (8.22)	16.82 (9.80)	−10.59	−5.09	<0.001	−1.14
STarT_Back_Total	0.61 (0.88)	2.45 (1.55)	−1.84	−6.08	<0.001	−1.37
GHQ_Total	8.35 (3.79)	9.25 (4.54)	−0.90	−0.93	0.353	−0.21

FAB: Fear-Avoidance Beliefs Questionnaire; GHQ: General Health Questionnaire.

**Table 3:** Multiple linear regression analysis for Schober’s test.

	Flexion				Extension			
	B (SE)	Beta	*t*	*p*-Value	B (SE)	Beta	*t*	*p*-Value
PAIN (Yes vs. No)	0.48 (0.23)	0.26	2.08	0.041	0.27 (0.23)	0.15	1.16	0.249
GHQ_Total	−0.05 (0.02)	−0.25	−2.39	0.019	0.01 (0.02)	0.04	0.31	0.755
FAB_Total	0.03 (0.01)	0.30	2.21	0.030	0.01 (0.01)	0.14	0.97	0.336
STarT_Back_Total	−0.09 (0.08)	−0.16	−1.10	0.276	−0.17 (0.08)	−0.32	−2.06	0.043

B: unstandardized regression coefficient. SE: standard error.

**Table 4:** Multiple linear regression analysis for side flexion.

	Right				Left			
	B (SE)	Beta	*t*	*p*-Value	B (SE)	Beta	*t*	*p*-Value
Pain (Yes vs. No)	−2.62 (1.22)	−0.28	−2.14	0.035	−2.22 (1.07)	−0.23	−2.07	0.041
GHQ_Total	0.01 (0.12)	0.01	0.12	0.908	0.10 (0.12)	0.10	0.85	0.399
FAB_Total	0.03 (0.06)	0.08	0.56	0.578	0.03 (0.06)	0.07	0.48	0.634
STarT-Back_Total	−0.12 (0.43)	−0.04	−0.27	0.787	−0.26 (0.44)	−0.09	−0.59	0.559

B: unstandardized regression coefficient. SE: standard error.

**Table 5:** Multiple linear regression analysis for rotation.

	Right				Left			
	B (SE)	Beta	*t*	*p*-Value	B (SE)	Beta	*t*	*p*-Value
Pain (Yes vs. No)	7.85 (3.87)	0.25	2.03	0.046	5.97 (4.24)	0.19	1.41	0.163
GHQ_Total	0.23 (0.40)	0.07	0.58	0.562	0.03 (0.41)	0.01	0.07	0.945
FAB_Total	−0.29 (0.21)	−0.20	−1.39	0.168	−0.21 (0.21)	−0.14	−0.97	0.334
STarT-Back_Total	−0.05 (1.46)	−0.01	−0.03	0.975	1.27 (1.48)	0.13	0.86	0.393

B: unstandardized regression coefficient. SE: standard error.

## Data Availability

No new data were created or analyzed in this study. Data sharing is not applicable to this article.
